# Clinical Profile, Arrhythmias, and Adverse Cardiac Outcomes in Emery–Dreifuss Muscular Dystrophies: A Systematic Review of the Literature

**DOI:** 10.3390/biology11040530

**Published:** 2022-03-30

**Authors:** Anna Chiara Valenti, Alessandro Albini, Jacopo Francesco Imberti, Marco Vitolo, Niccolò Bonini, Giovanna Lattanzi, Renate B. Schnabel, Giuseppe Boriani

**Affiliations:** 1 Cardiology Division, Department of Biomedical, Metabolic and Neural Sciences, University of Modena and Reggio Emilia, Policlinico di Modena, 41124 Modena, Italy; annachiaravalenti@gmail.com (A.C.V.); alessandro.albini91@gmail.com (A.A.); jacopo.imberti@hotmail.it (J.F.I.); marco.vitolo@unimore.it (M.V.); bonini.niccolo93@gmail.com (N.B.); 2 Clinical and Experimental Medicine PhD Program, University of Modena and Reggio Emilia, 41124 Modena, Italy; 3 CNR Institute of Molecular Genetics “Luigi Luca Cavalli-Sforza”, Unit of Bologna, 40136 Bologna, Italy; lattanzi@area.bo.cnr.it; 4 IRCCS Istituto Ortopedico Rizzoli, 40136 Bologna, Italy; 5 Department of Cardiology, University Heart and Vascular Center, 20246 Hamburg, Germany; r.schnabel@uke.de; 6 German Center for Cardiovascular Research (DZHK), Partner Site Hamburg/Kiel/Luebeck, 20246 Hamburg, Germany

**Keywords:** arrhythmias, atrial fibrillation, heart failure, implantable cardioverter–defibrillators, laminopathies, pacemaker, sudden cardiac death

## Abstract

**Simple Summary:**

Cardiolaminopathies portend an augmented risk of atrial and ventricular arrhythmias, thromboembolic events, ventricular dysfunction, and mortality. A high level of evidence on the clinical management of cardiac involvement in this setting is still lacking, and strong recommendations cannot be formulated. Our systematic review reported the incidence rates for the main cardiovascular outcomes in 1070 *LMNA* mutation carriers and 40 *EMD* mutation carriers, with a minimum follow-up of one-year. Our results stressed the importance of a structured follow-up aiming at the early recognition of atrial arrhythmias and the prevention of thromboembolic events. In patients with a need for pacing, cardioverter–defibrillator implantation should be considered due to the high burden of malignant ventricular arrhythmias.

**Abstract:**

Cardiolaminopathies are a heterogeneous group of disorders which are due to mutations in the genes encoding for nuclear lamins or their binding proteins. The whole spectrum of cardiac manifestations encompasses atrial arrhythmias, conduction disturbances, progressive systolic dysfunction, and malignant ventricular arrhythmias. Despite the prognostic significance of cardiac involvement in this setting, the current recommendations lack strong evidence. The aim of our work was to systematically review the current data on the main cardiovascular outcomes in cardiolaminopathies. We searched PubMed/Embase for studies focusing on cardiovascular outcomes in *LMNA* mutation carriers (atrial arrhythmias, ventricular arrhythmias, sudden cardiac death, conduction disturbances, thromboembolic events, systolic dysfunction, heart transplantation, and all-cause and cardiovascular mortality). In total, 11 studies were included (1070 patients, mean age between 26–45 years, with follow-up periods ranging from 2.5 years up to 45 ± 12). When available, data on the *EMD*-mutated population were separately reported (40 patients). The incidence rates (IR) were individually assessed for the outcomes of interest. The IR for atrial fibrillation/atrial flutter/atrial tachycardia ranged between 6.1 and 13.9 events/100 pts–year. The IR of atrial standstill ranged between 0 and 2 events/100 pts-year. The IR for malignant ventricular arrhythmias reached 10.2 events/100 pts–year and 15.6 events/100 pts–year for appropriate implantable cardioverter–defibrillator (ICD) interventions. The IR for advanced conduction disturbances ranged between 3.2 and 7.7 events/100 pts–year. The IR of thromboembolic events reached up to 8.9 events/100 pts–year. Our results strengthen the need for periodic cardiological evaluation focusing on the early recognition of atrial arrhythmias, and possibly for the choice of preventive strategies for thromboembolic events. The frequent need for cardiac pacing due to advanced conduction disturbances should be counterbalanced with the high risk of malignant ventricular arrhythmias that would justify ICD over pacemaker implantation.

## 1. Introduction

In 1966, Emery and Dreifuss for the first time reported on a family with X-linked, recessively inherited, slowly progressive skeletal muscle weakness and wasting; early contractures of the elbows, ankles and posterior neck; and cardiac disease [1]. The phenotype, age of onset and progression tendency differed individually. In the subsequent decades, genetic and physio-pathological studies allowed the understanding of the genetic heterogeneity of Emery–Dreifuss muscular dystrophy (EDMD) with the identification of an X-linked form in 1994 (EDMD1, caused by mutations in the *EMD* gene encoding emerin) with a prevalence of 1:100,000 [2], and an autosomal dominant form in 1999 (EDMD2, associated with mutations in the *LMNA* gene encoding A-type lamins) [3]. A few months later, forms of dilated cardiomyopathy with conduction system disorders without a skeletal muscle phenotype were identified [4]. Between 2000 and 2014, other nuclear envelope genes were linked to Emery–Dreifuss muscular dystrophy—EDMD4 and EDMD5—such as *SYNE1* and *SYNE2* encoding the nesprins [5,6], and *SUN1* and *SUN2* genes [7]. Moreover, mutations in the *FHL1* gene have been associated with another X-linked form called EDMD6 [8].

Emerin is an integral protein that concentrates in the inner nuclear membrane of cells and requires A-type lamins for its inner nuclear membrane localization. It is involved in several cellular processes, such as chromatin tethering, gene regulation, mitosis, nuclear assembly, and cellular signalling [9].

Lamins are the main nucleoskeletal components of the nuclear membrane, and account for both nuclear morphology and function. In mammalian cells, three genes are responsible for encoding seven proteins, classified as A-Type and B-Type lamins, but only mutations in A type lamins cause cardiomyopathy [10,11,12]. Lamins are involved in the determination of nuclear shape and size, in resisting deformation, and in anchoring elements of the nuclear envelope to their proper position [11]. Moreover, A-type lamins regulate the cellular response to mechanical, oxidative or replicative stress, chromatin dynamics, and the DNA damage response [13].

Mutations of lamins or their binding proteins have been related to a heterogeneous group of inherited disorders (collectively referred to as laminopathies), the phenotypic variety of which has powered deeper insights into their structural and functional role in nuclear architecture. Laminopathic nuclei appear misshapen, and cluster both in cultured muscle cells and in situ in skeletal and cardiac muscle [7,14,15,16]. According to the “structural hypothesis”, mutated lamins affect the mechanical properties of nuclei, and promote the aberrant localization of their binding partners. This effect may be particularly relevant in cells experiencing significant contractile forces, justifying the deleterious impact observed in tissues exposed to extreme mechanical stress, i.e., skeletal and cardiac muscles. The “gene regulation hypothesis” arises from the observation of lamins close to the sites of DNA and RNA processing, and in specific chromatin regions called lamina-associated domains (LADs), as well as from the finding that lamins may interact with transcription factors, and may regulate their nuclear import or activity [17,18,19,20,21]. These findings suggest that lamins may modulate gene expression according to tissue-specific patterns and variably during development [22,23,24,25]. In particular, it has been recently shown that SCN5A sodium channels are affected by the K219T *LMNA* mutation, a condition that contributes to the cardiac conduction defects which are typical of cardiolaminopathies [26].

In Emery–Dreifuss muscular dystrophy, cardiac manifestations may include life-threatening bradyarrhythmias, either of sinus and atrial origin (sino-atrial blocks, atrial standstill (ASS)) or atrioventricular blocks (AVB) (up to third-degree atrioventricular blocks), ventricular tachyarrhythmias, sudden cardiac death (SCD), and dilated cardiomyopathy (DCM) with heart failure (HF) of variable degrees [27,28,29]. Phenotypic manifestation in terms of cardiac and striated muscle involvement is partially age-related but is extremely heterogeneous, such that the same patient can manifest arrhythmic and skeletal features at the same time, or one manifestation could be present earlier than the other, or could even not become clinically relevant for a long time [28]. This heterogeneity in clinical manifestations and the substantial lack of high-quality evidence from controlled trials imply that a standardized approach in risk stratification and therapeutic choices is hard to establish, and the guideline recommendations rely on expert consensus.

Given the prognostic significance of cardiac manifestations in patients with nuclear proteins mutations, we focused on *LMNA*-related diseases in adult patients, and performed a systematic review of the available evidence in order to assess the incidence of the most relevant cardiovascular outcomes in this specific setting.

## 2. Materials and Methods

The present systematic review was conducted according to the Preferred Reporting Items for Systematic Reviews and Meta-Analysis (PRISMA) recommendations and checklist [30]. We performed an extensive literature search using a combination of two major medical literature databases: Pubmed and Embase. Our search was restrained to full-text articles which were available up to 1 September 2021. No restriction was applied to the study type or design.

The search strategy used a combination of the following terms and their MeSH: ((“Emery–Dreifuss”) OR (“LMNA”) OR (“Lamin A/C”) OR (“Laminopathies”) OR (“Muscular Dystrophies”) OR (“lamin”) OR (“Laminopathy”)) AND ((“Atrial Fibrillation”) OR (“defibrillator”) OR (“Pacemaker”) OR (“Arrhythmias”) OR (“Heart Failure”) OR (“Death, Sudden, Cardiac”)). The whole syntax is shown in the Supplementary Materials (Table S1).

All of the studies assessing the incidence of structural cardiomyopathy, atrial arrhythmias, conduction disorders, malignant arrhythmias, heart transplantation (HT) and mortality (both all-cause and cardiovascular) were included.

We used a two-step method for the study selection. Two investigators (A.A. and A.C.V.) independently screened the records for eligibility based on English language, titles, and abstracts. Non-pertinent publications, genetics studies on cell cultures or animal models, and case reports, editorials, meeting abstracts, research letters and reviews were excluded. Second, any original study satisfying the following criteria was considered eligible for inclusion: (i) sustained arrhythmic events (both supraventricular and ventricular), the need for device implantation (pacemaker (PM) or implantable cardioeverter-defibrillator (ICD)), appropriate ICD intervention, stroke, HT, and mortality among the study outcomes; (ii) cohorts of at least 15 subjects; and (iii) a minimum follow-up duration of one year. A senior reviewer (G.B.) independently analysed the study selection and the data extraction process. When two or more studies analysed the same population, we chose to include the one with the largest cohort and the most comprehensive data (see Table S2 for the list of studies excluded due to overlapping cohorts). Discrepancies were resolved by consensus.

When available, the following data were reported from each study: the number of patients enrolled, their sex, their mean or median age, the mean or median follow-up period, neuromuscular involvement, median or mean left ventricular ejection fraction (LVEF) values, the percentage of patients with reduced LVEF, NYHA class, sinus node dysfunction, conduction disturbances, and atrial arrhythmias. The outcomes from subgroups with *EMD*-related mutations were reported separately, when available.

In our analysis, we analysed the initial point prevalence, the incidence during the follow-up period, and the incidence rate (IR) of the main arrhythmic events reported in the studies: atrial arrhythmias (atrial fibrillation (AF), atrial flutter (AFL), atrial tachycardia (AT), and ASS), major ventricular arrhythmias (composite outcome comprising sustained ventricular tachycardia (VT), ventricular fibrillation (VF), SCD, and appropriate ICD intervention), and clinically relevant conduction disturbances. We also analysed the incidence of PM and ICD implantation, stroke, death (all-cause and cardiac) and HT.

The methodological quality of the studies were evaluated by two investigators independently (A.A. and A.C.V.) using the Newcastle–Ottawa scale in order to assess the risk of bias for observational studies [31]. The studies were of sufficient quality when they scored ≥5.

The continuous variables are reported as the mean or median, and the categorical variables are reported as the number and percentage. The IR for the outcomes of interest were calculated considering the cumulative incidence of the events in the population at risk, and assuming the median follow-up reported in the study as the effective follow-up period for each patient. The IR was expressed for 100 pts–year. When the effective number of incident events could not be inferred, the prevalence at the end of the follow-up was used as a comparison with the baseline data. In case of data on a subgroup in the study, it is reported in the tables. For the analysis of the ICD intervention IR, when the follow-up for patients with an ICD was not specified, we used the final prevalence of ICD implantation and the median follow-up time to calculate the pts–year. Due to the relevant heterogeneity among the studies in the systematic review in terms of design and reported outcomes, an aggregate data meta-analysis could not be performed. The studies’ outcomes were collected and reported according to these definitions, unless otherwise specified. All of the analysis was performed using Microsoft Excel software (version 16.56).

## 3. Results

### 3.1. Literature Search and Study Screening

**Figure 1:**
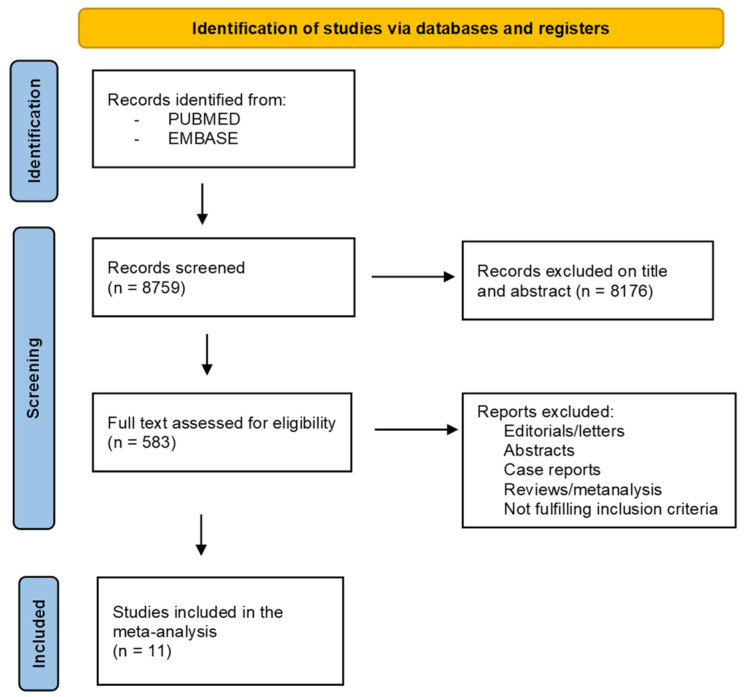
Study selection process (PRISMA flow diagram).

A systematic search of electronic databases identified a total of 8759 articles, after removing duplicates. Of these, 8176 were excluded based on their title and abstract. The remaining 583 were evaluated through full-text revision. Finally, 11 articles were included in the analysis [27,28,29,32,33,34,35,36,37,38,39], as shown in Figure 1.

Among the included articles, two studies were prospective [33,38], seven were retrospective [27,28,29,32,34,35,39], and two were based on both prospective and retrospective data [36,37]. The studies ranged from 2003 to 2021, while the sample size ranged from 18 to 269 patients. Overall, 1110 patients were included in the present analysis. Among the studies, we identified a cohort of *LMNA* mutation carriers and a cohort of *EMD* mutation carriers. The study design, the baseline characteristics of the patients included in the studies, and the main findings are summarized in Table 1.

### 3.2. Methodological Quality of the Data

Among the selected studies, the methodological quality was high, with all of the studies having a Newcastle–Ottawa scale score ≥5. The mean score was 6.1 ± 0.9 SD, as reported in Table 2.

### 3.3. Atrial Arrhythmias

Baseline reports on the personal history of previous atrial arrhythmias were available for almost all of the selected studies. The point prevalence of atrial arrhythmias at the baseline ranged from 12.5% to 63% in the *LMNA* patient cohort, and from 10% to 73.3% in the *EMD* patient cohort. A very small proportion of the studies reported on the prevalence of sinus rhythm dysfunction or sinoatrial block (SSS-SAB) at the baseline; when reported, it ranged from 10% to 25% of the patients. The IR of AF/AFl/AT ranged between 6.1 events/100 pts–year and 13.9 events/100 pts–year. The IR for SSS/SAB was assessable only for the population studied by Boriani et al. [27]. In this cohort, the IR for SSS/SAB was 8.6 events/100 pts–year among the *LMNA* group, and 3.5 events/100 pts–year in the *EMD* group. The IR of ASS ranged between 0 and 2 events/100 pts–year in the *LMNA* cohort, and reached 3.3 events/100 pts–year in the *EMD* cohort (Table 3).

### 3.4. Ventricular Arrhythmias and Sudden Cardiac Death

At the baseline, a small group of patients from the *LMNA* cohort experienced sustained ventricular tachyarrhythmias (SVT), whereas no one in the *EMD* cohort reported a personal history of these rhythm disturbances. During the follow-up, the IR for the composite outcome of malignant ventricular arrhythmias (MVA) reached 10.2 events/100 pts–year and 15.6 events/100 pts–year for the appropriate ICD interventions. On the other hand, in the small *EMD* cohort, only two patients experienced MVA during the follow-up. Consequently, the IR of ICD implantation in the *LMNA* cohort overstepped the implantation rate in the *EMD* cohort (Table 4).

### 3.5. Conduction Disturbances

The prevalence of clinically significant conduction disturbances (AVB II, III) in the two groups ranged from 12.5% to 46.6% (67% when considering I–II–III grade AVB) at the baseline. The IR for II–III AVB ranged between 3.2 and 7.7 events/100 pts–year in the *LMNA* group, and was 2.3 events/100 pts–year for the *EMD* group in a single study [27]. The IR of PM implantation was slightly higher in the *EMD* (Table 5).

### 3.6. Thromboembolic Events and Stroke

In the total cohort of 1021 patients, 56 thromboembolic events/strokes occurred. The IR of thromboembolic events ranged from 0.3 events/100 pts–year to 8.9 events/100 pts–year (Table 6).

### 3.7. Ventricular Dysfunction, Heart Failure and Heart Transplantation

Across the studies, data on left ventricular dysfunction (LVD)/DCM were differently reported, such that the representation of the two cohorts at the baseline resulted in a problematic interpretation. HF with advanced symptoms (NYHA functional class ≥ III–IV) was reported in 0–19.3% of the patients at the baseline, with progression during the follow up. The severity of HF in some cases led to HT. During the follow-up, the IR for HT ranged from 0.1 event/100 pts–year to 10 events/100 pts–year (Table 7).

### 3.8. All-Cause Death and Cardiovascular Mortality

The IR for all-cause death during the follow-up in the two cohorts ranged from 0.6 events/100 pts–year to 4.8 events/100 pts–year. The majority of these deaths were related to SCD or advanced HF. The comparison between the two cohorts showed a greater incidence of death in the total *LMNA* cohort compared to the small *EMD* group (Table 8).

## 4. Discussion

The study of the natural history of rare diseases has inherent limitations related to small sample sizes, variable follow-up durations, and focusing on different clinical outcomes [40].

**Figure 2:**
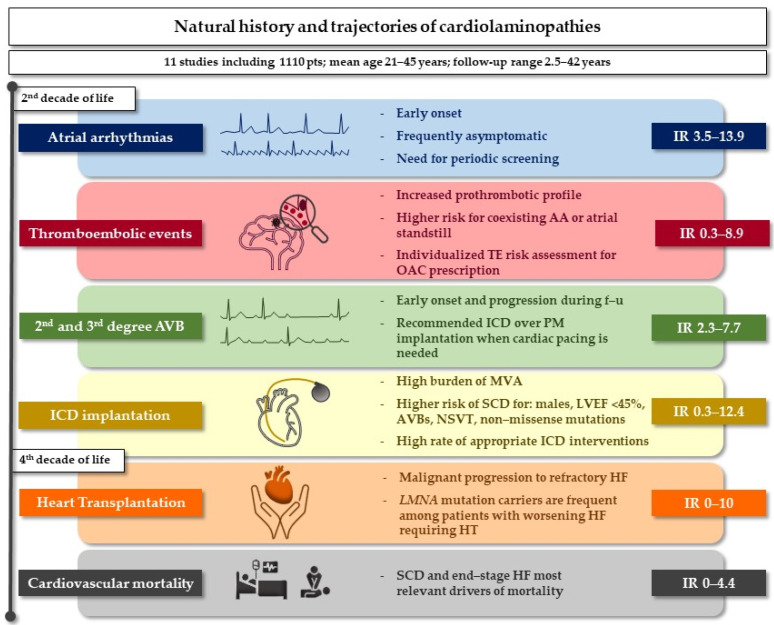
Natural history of cardiolaminopathies and main findings. Legend: AA, atrial arrhythmias; AVB, atrioventricular block; DCM, dilated cardiomyopathy; HF, heart failure; HT, heart transplantation; ICD, implantable cardioverter–defibrillator; IR, incidence rate; LVEF, left ventricular ejection fraction; MVA, malignant ventricular arrhythmias; NSVT, non-sustained ventricular tachycardia; OAC, oral anticoagulant; PM, pacemaker; SCD, sudden cardiac death.

Laminopathies are rare diseases that require careful clinical assessment, and the natural history of cardiac involvement has been the object of a series of reports. In view of the dispersion of the data published in the literature, we performed a systematic review of the main cardiac outcomes in patients with laminopathies. Our main findings depict the real-life impact of cardiac involvement in a cohort of 1110 adult patients during a follow-up time that ranges from 2.5 to 42 years (Figure 2).

### 4.1. Atrial Arrhythmias

Atrial arrhythmias are frequent in our analysis, from the second decade of life onwards, with the peculiar predisposition to AF and brady-arrhythmias, two types of arrhythmias which are absolutely unusual in this age group [41,42]. Atrial cardiomyopathy is a nosological entity that subtends a broad variety of pathophysiological mechanisms, leading to various histological changes [43]. The atrial remodelling promotes the onset, maintenance, and progression of AF, and has been related per se to an increased risk ok ischemic events [44]. In *LMNA*-related cardiomyopathy, myocardial fibrosis and fibro-lipomatosis are the main histological features of atrial remodelling, and are held responsible of both atrial arrhythmogenesis and conduction disturbances [45,46]. However, previous studies also highlighted a higher rate of early afterdepolarizations, spontaneous depolarization or quiescence, and the spontaneous onset of tachyarrhythmias in *LMNA* mutated cardiomyocytes compared to control cells [46]. These data stress that atrial arrhythmias in laminopathies are the early manifestation of an atrial cardiomyopathy characterized by the profound derangement of atrial structure and function, which may lead to important clinical consequences, including ischemic stroke at a relatively young age [44,47].

In the general population, the prevalence of AF is expected to be significantly lower among young patients than in older people (0.05% in patients younger than 30 years, up to 10% in those older than 80) [48]. The estimated age-adjusted IR for 2010 was 0.07 in men of all ages worldwide (77.5 per 100,000 person-years) and 0.05 in women (59.5 per 100,000 person-years) [49]. Additionally, young patients used to be particularly symptomatic due to the high ventricular response rates which are usually sustained by atrial tachycardias.

In the case of pathogenic *LMNA* mutations, patients show a very premature onset of atrial arrhythmias from the second decade of life onwards, independently from the degree of cardiac involvement, and even before the cardiomyopathy becomes manifest. As shown in our results (Table 3), the prevalence of atrial arrhythmias is visibly remarkable even at the very first cardiological assessment, and then further increases for each cohort during the follow-up. An interesting observation is that atrial arrhythmias are frequently asymptomatic or only mildly symptomatic in *LMNA*-mutated patients, due to normal/low mean heart rates, possibly leading to delayed recognition or under-recognition [27,50]. Considering that, in our analysis, the IR for atrial arrhythmias ranged from 5.4 up to 12.9 (100 pts–year), periodic ECG screening or extended monitoring should be promoted, also using wearable devices, independently of patients’ age and symptoms [51,52].

Moreover, even if genetic testing is not routinely applied in AF patients, recent data highlighted that early-onset arrhythmias or a strong family history of AF may be related to pathogenic variants of genes linked to cardiomyopathies, including *LMNA* [53].

### 4.2. Thromboembolic Risk

A higher thromboembolic risk has been reported among *LMNA* mutation carriers compared to the general population of the same age (IR ranging between 0.3 and 8.9 events/100 pts–year in our cohort). Thromboembolic events often originate from cardioembolic sources and relate to atrial arrhythmias or ASS, or to the underlying atrial cardiomyopathy [54,55,56]. Among *LMNA* mutation carriers, AF per se carries nine-fold increased odds for ischemic stroke (OR 9.2, 95%CI 1.1–74.7) [35], which actually corresponds to a higher risk compared to that reported for conventional AF patients, either symptomatic or asymptomatic [52,57,58]. The final stage of electrical disarray is an ASS that also subtends mechanical inactivity and promotes thrombus formation [27,50]. Nevertheless, according to our results (Table 3), the IR for ASS are low (ranging from 0 to 2/ 100 pts–years) among the *LMNA*-mutated cohort. Although data on ASS are poorly reported by the included studies, ASS seems to be an infrequent and late condition that may have an important impact on the thromboembolic profile. However, not only these conditions but also marked sinus bradycardia and advanced conduction disturbances contribute to the higher thromboembolic risk profile of *LMNA*-mutated patients. It is noteworthy that, in the population studied by Kumar et al., two out of ten patients diagnosed with strokes did not report any atrial arrhythmia during the entire follow-up [28]. Van Rijsingen et al. suggested that an *LMNA* mutation, per se, is related to a prothrombotic profile independently of coexisting atrial arrhythmias, structural heart disease or device implantation. According to their results, the risk of thromboembolic events is almost five-fold higher in the case of *LMNA* mutations (HR 4.8, 95% CI 2.2–10.6) and remains significant for the individual components of arterial and venous complications (HR 5.6, 95%CI 2.3–14 and HR 6.5, 95%CI 1.7–25.8, respectively) [34].

This observation should guide clinical decisions on therapeutic management, independently from conventional thromboembolic scoring systems [57]. Because atrial arrhythmias appear at a young age, and independently of the coexistence of systolic dysfunction, most patients would not be eligible for oral anticoagulants according to conventional thromboembolic risk scores (CHADS_2_, CHA_2_DS_2_VASc). However, considering the natural history of cardiolaminopathies, it appears reasonable to introduce oral anticoagulants in *LMNA* mutation carriers that develop atrial arrhythmias even if data on the efficacy of antiplatelets/oral anticoagulants are scarce.

### 4.3. Conduction Disturbances and Malignant Ventricular Arrhythmias

The burden of MVA is impressive, especially in the *LMNA*-mutated cohort; the implantation of the ICD resulted in device activation in 24 to 52.4% of ICD carriers. In parallel to this finding, clinically significant conduction disturbances (AVB II-III) are very common after the very first clinical evaluation (ranging between 11.7 and 67% at the baseline assessment in the *LMNA* group), and progress to more advanced forms during the follow-up, with the need for cardiac pacing. The risk of ventricular arrhythmias and the growing evidence on the role of ICD in the prevention of SCD in this setting markedly conditions the choice for implantable devices with the preference for ICD over PM when the need for cardiac pacing occurs [57]. In a pivotal metanalysis by Van Berlo et al., the authors underlined for the first time that, even if a significant number of patients with *LMNA* mutations underwent PM implantation for conduction disturbances, this intervention did not alter the risk of SCD in *LMNA* mutation carriers [59]. Moreover, when an ICD was implanted in patients with conventional criteria for pacing, an appropriate intervention occurred in 42% and 52% of patients within 3 and 5 years, respectively [33,58]. Moving from this evidence, specific indications for patients with an *LMNA* mutation were provided in the last version of European guidelines on pacing [58,60].

However, the proper risk stratification of SCD in non-ischemic DCM is still a matter of debate. In fact, despite the current recommendations identifying reduced LVEF as the main prognostic stratifier in the choice for ICD implantation, any survival benefit was highlighted in the DANISH trial among non-ischaemic DCM patients who underwent prophylactic ICD implantation [61]. Previously, among 158 patients with idiopathic DCM, Gatzoulis et al. observed a higher rate of appropriate ICD intervention or documented SCD when sustained ventricular arrhythmias were induced during programmed ventricular stimulation. Interestingly, this finding was noted even in the subset of patients with LVEF > 35% [62]. More recently, Escobar-Lopez et al. underlined the prognostic role of genetic variants in a large cohort of 1005 patients with genotyped DCM. A considerably higher risk of for arrhythmic events was found in the subgroup with mutations for the nuclear envelope, in which the *LMNA*-related DCM prevailed compared to other functional gene groups or genotype negative patients. A similar trend was observed even in mutation carriers with LVEF > 35% [63].

Given the need for further evidence, these results highlight that several factors other than LVEF act in defining the risk for arrhythmic events among non-ischaemic DCM patients. Considering the great heterogeneity of this cohort, a more comprehensive and genotype-guided approach would be advisable for the prevention of SCD.

In the studies included in our analysis, some risk factors for the development of life-threatening ventricular arrhythmias were identified, e.g., the ejection fraction at the baseline < 45%, male sex, non-missense mutations, non-sustained ventricular tachycardia (NSVT) at presentation, and AVB. The guidelines suggest the implementation of a recently developed and validated module to estimate the ventricular arrhythmic risk in *LMNA* mutation carriers [64].

### 4.4. Systolic Dysfunction and Heart Transplantation

Lamin-related cardiomyopathy seems to be particularly malignant compared to other aetiologies of DCM, with the frequent need for HT [65]. Our data highlight this information, which has important practical implications for the clinical management of HF in *LMNA* mutation carriers. No specific echocardiographic features have been described in this setting. Thus, the clinical suspicion is mainly driven by the family history, together with the coexistence of signs and symptoms of neuromuscular disease. However, it must be underlined that the structural cardiac disease progresses independently from other districts’ involvement, and may also precede them. Sinus node dysfunction or conduction abnormalities frequently advance the onset of LV dilation, and the decrease in contractility. Remarkably, once conduction disturbances have been assessed, MVA are common, independently of LVEF, and are responsible for a higher risk of SCD [66,67,68]. Moreover, *LMNA*-related cardiomyopathies typically evolve to refractory end-stage heart failure with the need for HT [65].

The data on the baseline prevalence of systolic dysfunction were not homogeneous in the included studies due to the different definitions adopted by the authors. Similarly, the baseline and final functional assessment (NYHA class) were not systematically collected in most of the studies. When available, the prevalence for NYHA class ≥III–IV ranged between 0% and 19.3% at the first clinical evaluation, and rose to 34% at the end of the follow-up. The IR for HT reached 10 events/100pts–year in our cohort. According to Hasselberg et al., *LMNA* mutation carriers were more frequently represented among the 79 non-ischaemic DCM undergoing HT than among the general population of non-ischaemic DCM (6.5% vs. 1.5%, respectively), underscoring the poor prognosis of *LMNA*-related cardiomyopathies [29]. As was reported in a previous metanalysis exploring genotype–phenotype association in DCM, patients with *LMNA*-related DCM showed the highest frequency of HT compared to other mutated-gene groups (up to 27%), with a mean age of 41.4 at the time of HT. However, the reported mean LVEF in the *LMNA*-group was almost 35%, which was slightly higher with respect to other groups’ mean values [65]. It is noteworthy that HF appears, in general, to be a clinical manifestation that—in most of the patients and case series—occurs later compared to arrhythmic manifestations [28,57]. The prognostic impact of cardiac involvement in laminopathies is even clearer when analysing the impact of cardiac death with respect to all-cause death in our population. As was predictable, SCD and end-stage HF were the most relevant drivers of mortality in the entire cohort.

### 4.5. Limitations

Our study has several limitations. Due to its observational design as a systematic review of observational studies, it may be influenced by publication bias because of the possibility that some data were not published or were missed from the literature search. The heterogeneity of the analysed studies is the most relevant limitation, and prevented us from performing an aggregate data meta-analysis. Considering that the *EMD*-mutated population is poorly represented in our cohort, the comparison with the *LMNA*-mutated group did not highlight relevant differences in terms of outcomes. Moreover, the differences in the baseline characterization of the population, especially when considering LV dysfunction, was a significant limitation for the interpretation of arrhythmic outcomes, ICD implantation indications and interventions. Our systematic review was focused on cardiac events, and we did not consider neurological and respiratory impairment, which may be an important determinant of the quality of life and outcome in some patients [36]. Furthermore, we did not address the assessment of treatments that have been proposed for these rare diseases, which have been recently systematically analysed [69]. Overall, this is one of the few, comprehensive, contemporary summaries on the rare laminopathies in relation to their cardiac manifestations.

## 5. Conclusions

Cardiac involvement in laminopathies such as EDMD is frequent and heterogeneous, and has an important prognostic significance. The early identification of atrial arrhythmias in young patients could be crucial, especially with respect to the thromboembolic risk that these mutations portend. The need for pacing is high during the natural history of the disease, and our data strengthen the concept that ICDs should be preferred over PMs in this setting, especially when risk factors are present. Lastly, *LMNA*-related cardiomyopathy carries a substantial risk of end-stage HF development even in young patients with a high proportion of HT. Improved methods for risk stratification should be prospectively validated in order to appropriately target innovative treatments, such as gene therapy.

## Figures and Tables

**Table 1:** Study design and baseline characteristics.

Study, Year	Study Design	Population (n)	Age (y), Median or Mean ± SD	Median F-U (y)	Women, n (%)	Unaffected or Asymptomatic, n (%)	Neuromuscular Involvement, n (%)	Main Findings
***LMNA*** **mutation carriers**								
Boriani et al. [27], 2003	Retrospective	8 (18 total cohort)	29.5	9.5 ± 9	2 (25)	1 (12.5)	6 (75)	Pts with *EMD* mutations are at great risk to develop AF, AFL, bradycardia, ASS and stroke
Van Rijsingen et al. [32], 2012	Retrospective	269	36	3.5	121 (45)	56/248 (23)	41/198 (21)	Among *LMNA* mutation carriers, male sex, EF < 45%, NSVT and non-missense mutations portend a greater risk of MVA
Anselme et al. [33], 2013	Prospective	47	38 ± 11	7.9 (5.1 ICD carriers)	21 (45)	N/A	[isolated nm involv.] 18 (38)	MVA are frequent in *LMNA* carriers with cardiac conduction disorders irrespective of EF
Van Rijsingen et al. [34], 2013	Retrospective	76	45	42 ± 12 (mean ± SD)	35 (46)	N/A	25 (33)	*LMNA* mutation is an independent predictor of arterial and venous TE
Kumar et al. [28], 2016	Retrospective	122	41	7	52 (43)	18 (9)	18 (15)	*LMNA*-related heart disease is associated with a high frequency of phenotypic progression and index phenotype does not predict adverse events
Hasselberg et al. [29], 2018	Retrospective	79	42 ± 16	7.8	36 (46)	N/A	N/A	Among DCM, the prevalence of *LMNA* mutation is 6.2% with high penetration in asymptomatic young genotype-positive members. *LMNA* carriers have a high incidence of HT.
Nakajima et al. [35], 2018	Retrospective	110	43 ± 15	5	42 (38)	N/A	N/A	Several cardiac presentations are age-related in *LMNA*-related heart disease, LVD is the only predictor for mortality
Peretto et al. [36], 2019	Prospective/ Retrospective	164	38	10	84 (51)	N/A	104 (63)	Many *LMNA* mutation carriers develop neurological disease in their 30s and cardiac manifestation in the next decade
Ditaranto et al. [37], 2019	Prospective/ Retrospective	40	39	2.5	18 (45)	N/A	14 (35)	Pts with neuromuscular presentation have an earlier cardiac involvement (from AF and/or AVB to cardiomyopathy)
Marchel et al. [38], 2021	Prospective	15 (45 total cohort)	26	11	11 (73)	N/A	15 (100)	Atrial arrhythmias are common in *EMD*/*LMNA* mutation carriers; they occur earlier in *EMD* pts. VA are common (60%) and earlier in *LMNA* compared to the *EMD* group
Barriales-Villa et al. [39], 2021	Retrospective	140	42.8 M, 38 F	5 (probands), 3 (relatives)	69 (49.3)	N/A	34 (24.3)	Among *LMNA* mutation carriers, NSVT and EF < 45% were the only independent predictors of MVA
***EMD*** **mutation carriers**								
Boriani et al. [27], 2003	Retrospective	10 (18 total cohort)	24.5 (affected males)	16	3 (30)	0 (0)	6 (60)	Pts with *EMD* mutations are at great risk to develop AF, AFL, bradycardia, ASS and stroke
Marchel et al. [38], 2021	Prospective	30 (45 total cohort)	21	11	6 (20)	N/A	30 (100)	Atrial arrhythmias are common findings in *EMD*/*LMNA* mutation carriers, they occurred earlier in *EMD* pts. VA were very common (60%) in *LMNA* and occurred definitely earlier compared to the *EMD* group

Legend: AF, atrial fibrillation; AFL, atrial flutter; ASS, atrial standstill; AVB, atrio-ventricular block; DCM, dilated cardiomyopathies; EF, ejection fraction; *EMD*, Emery–Dreifuss; F-U, follow-up; HT, heart transplantation; ICD, implantable cardiac defibrillator; *LMNA*, lamin A/C; LVD, left ventricular dysfunction; MVA, malignant ventricular arrhythmias; NSVT, non-sustained ventricular tachycardia; Pts, patients; SD, standard deviation; TE, thromboembolic events; VA, ventricular arrhythmias; y, years.

**Table 2:** Newcastle–Ottawa quality assessment for the cohort studies. “*” stands for 1 point in the specific section.

Study	Selection	Comparability	Outcome	Total
Boriani et al. [27], 2003	**	-	***	5
Van Rijsingen et al. [32], 2012	**	**	***	7
Anselme et al. [33], 2013	**	**	***	7
Van Rijsingen et al. [34], 2013	**	**	***	7
Kumar et al. [28], 2016	**	-	***	5
Hasselberg et al. [29], 2018	**	**	***	7
Nakajima et al. [35], 2018	**	**	***	7
Peretto et al. [36], 2019	**	-	***	5
Ditaranto et al. [37], 2019	**	-	***	5
Marchel et al. [38], 2021	**	-	***	5
Barriales-Villa et al. [39], 2021	**	**	***	7

**Table 3:** Atrial arrhythmias.

	Population (n)	Age (y), Median or Mean ± SD	AF, AFL, AT, n (%)	SSS/SAB, n (%)	ASS, n (%)	Median F-U (y)	AF, AFL, AT, n (%)	SSS/SAB, n (%)	ASS, n (%)	IR AF/AFl/AT	IR SSS/SAB	IR ASS
			Baseline Prevalence				Incident Events or Final Prevalence					
***LMNA*** **mutation carriers**												
Boriani [27], 2003	8	29.5	1 (12.5)	0 (0)	N/A	7	3 (42.8)	3 (60)	1 (12.5)	6.1	8.6	1.8
Van Rijsingen IA [32], 2012	269	36	86/239 (36)	N/A	N/A	3.5	N/A	N/A	N/A	N/A	N/A	N/A
Anselme [33], 2013	47	38±11	12 (26)	N/A	N/A	7.9	31 (88.6)	N/A	N/A	11.2	N/A	N/A
Van Rijsingen [34], 2013	76	45	48 (63)	N/A	N/A	42 ± 12 (mean ± SD)	N/A	N/A	N/A	N/A	N/A	N/A
Kumar [28], 2016	122	41 ± 14	52 (42.7)	N/A	N/A	7	62 (88.6)	N/A	N/A	12.7	N/A	N/A
Hasselberg [29], 2018	79	42 ± 16	N/A	N/A	N/A	7.8	* 48 (no population at risk)	N/A	N/A	N/A	N/A	N/A
Nakajima [35], 2018	110 baseline/90 end of f-U	43 ± 15	31 (34.4)	27/110 (25)	N/A	5	27 (45.7)	30 (no population at risk)	N/A	9.2	N/A	N/A
Peretto [36], 2019	164	38	19/137 (14)	N/A	N/A	10	103 (no population at risk)	13 (no population at risk)	N/A	N/A	N/A	N/A
Ditaranto [37], 2019	40	39	17 (42.5)	4 (10)	N/A	2.5	8 (34.8)	N/A	2 (5)	13.9	N/A	2
Marchel [38], 2021	15	26	N/A	N/A	0 (0)	11	10 (66.6)	N/A	0 (0)	N/A	N/A	0
Barriales-Villa [39], 2021	140	40.4	42 (30)	N/A	N/A	3.8	N/A	N/A	N/A	N/A	N/A	N/A
***EMD *** **mutation carriers**												
Boriani [27], 2003	10	24.5 (affected males)	1 (10)	1 (10)	N/A	16	5 (55.5)	4 (44.4)	4 (40)	3.5	3.5	2.5
Marchel [38], 2021	30	21	22 (73.3)	N/A	3 (10)	11	* 11 (36.6)	N/A	11 (40.7)	N/A	N/A	3.7

Legend: AF, atrial fibrillation; AFL, atrial flutter; ASS, atrial standstill; AT, atrial tachycardia; IR, incident rate; SAB, sinoatrial block; SSS, sick sinus syndrome; y, years. * Prevalence at the end of the follow-up.

**Table 4:** Ventricular arrhythmic events.

	Population (n)	Age (y), Median or Mean ± SD	SVT, n (%)	Median F-U (y)	F-U ICD Carriers	MVA, n (%)	ICD Implantation, n (%)	ICD Appropriate Intervention, n (%)	IR MVA	IR ICD Implantation	IR ICD Intervention
			Baseline Prevalence			Incident Events					
***LMNA *** **mutation carriers**											
Boriani [27], 2003	8	29.5	N/A	7	N/A	N/A	N/A	N/A	N/A	N/A	N/A
Van Rijsingen IA [32], 2012	269	36	N/A	3.5	2.1	53 (19.7)	117 (43.5) [primary prevention 107 (39.8); secondary prevention 10 (3.7)]	28/117 (24)	5.6	12.4	11.4
Anselme [33], 2013	47	38 ± 11	N/A	7.9	5.1	14 (29.8)	21 (44.6)	11/21 (52.4)	3.8	5.7	10.3
Van Rijsingen [34], 2013	76	45	N/A	42 ± 12 (mean ± SD)	N/A	N/A	N/A	N/A	N/A	N/A	N/A
Kumar [28], 2016	122	41 ± 14	21 (17.2)	7	7	39 (32)	59 (48.3)	29/58 (50)	4.6	6.9	5.2
Hasselberg [29], 2018	79	42 ± 16	N/A	7.8	7.8	14 (17.7)	49 (62)	N/A	2.3	8	N/A
Nakajima [35], 2018	110 baseline/90 end of f-U	43 ± 15	VT + VF 21/110 (19)	5	5	46 (51.1)	44 (48.9)	12/44 (27.3)	10.2	9.8	5.2
Peretto [36], 2019	164	38	2/137 (1.5)	10	N/A	32 (19.5)	N/A	N/A	2	N/A	N/A
Ditaranto [37], 2019	40	39	N/A	2.5	2.5	SVT/storm 7 (17.5)	10 (25)	7/18 (38.9)	7	10	15.6
Marchel [38], 2021	15	26	VT 0 (0)	11	11	VT 2 (13.3)	9 (60)	N/A	1.2	5.5	N/A
Barriales-Villa [39], 2021	140	40.4	0 (0)	3.8	3.8	24 (17.1)	62 (44.3)	17/62 (27.4)	4.5	11.7	6.9
***EMD *** **mutation carriers**											
Boriani [27], 2003	10	24.5 (affected males)	N/A	16	N/A	N/A	N/A	N/A	N/A	N/A	N/A
Marchel [38], 2021	30	21	0 (0)	11	N/A	2 (6.7)	1 (3.3)	N/A	0.6	0.3	N/A

Legend: F-U, follow-up; ICD, implanted cardiac defibrillator; IR, incidence rate; MVA, malignant ventricular arrhythmias; SVT, sustained ventricular tachycardia; VF, ventricular fibrillation; VT, ventricular tachycardia; y, years.

**Table 5:** Conduction disturbances.

	Population (n)	Age (y), Median or Mean ± SD	2nd–3rd Degree AVB, n (%)	SSS, n (%)	Median F-U (y)	2nd–3rd Degree AVB, n (%)	PM Implantation, n (%)	IR 2nd-3rd AVB	IR PM Implantation
			Baseline Prevalence			Incident Events or Final Prevalence			
***LMNA *** **mutation carriers**									
Boriani [27], 2003	8	29.5	1 (12.5)	N/A	7	2 (25)	3 (37.5)	7.1	5.4
Van Rijsingen IA [32], 2012	269	36	114/244 (47), 1st, 2nd, 3rd AVB	N/A	3.5	N/A	N/A	N/A	N/A
Anselme [33], 2013	47	38 ± 11	21 (45), significant conduction disorders **	N/A	7.9	33 (no population at risk)	N/A	N/A	N/A
Van Rijsingen [34], 2013	76	45	51 (67), LMNA 1st, 2nd, 3rd AVB	N/A	42 ± 12 (mean ± SD)	N/A	N/A	N/A	N/A
Kumar [28], 2016	122	41 ± 14	18 (15.4)	N/A	7	27 (26)	N/A	3.7	N/A
Hasselberg [29], 2018	79	42 ± 16	N/A	N/A	7.8	* 51 (no population at risk), 1st, 2nd, 3rd AVB	N/A	N/A	N/A
Nakajima [35], 2018	110 baseline/90 end of f-U	43 ± 15	33 (36.7)	27/110 (25)	5	22 (38.6)	11 (12.2)	7.7	2.4
Peretto [36], 2019	164	38	16/137 (11.7)	N/A	10	75 (no population at risk)	N/A	N/A	N/A
Ditaranto [37], 2019	40	39	15 (37)	4 (10)	2.5	2 (8)	4(10)	3.2	4
Marchel [38], 2021	15	26	7 (46.6)	N/A	11	N/A	7 (46)	N/A	4.2
Barriales-Villa [39], 2021	140	40.4	34 (24.3)	N/A	3.8	N/A	36 (25.7)	N/A	6.8
***EMD *** **mutation carriers**									
Boriani [27], 2003	10	24.5 (affected males)	2 (20)	1 (10)	16	2 (25)	7 (70)	2.3	4.4
Marchel [38], 2021	30	21	14 (46.7)	ASS 3 (10)	11	N/A	23 (76.6)	N/A	7

* Prevalence at the end of the follow-up. ** Significant conduction disorders (PR > 240 ms, bradycardia, LBBB, NSVT, PM carriers). Legend: ASS, atrial standstill; AVB, atrioventricular block; IR, incidence rate; LBBB, left bundle branch block; NSVT, non-sustained ventricular tachycardia; PM, pacemaker; SSS, sick sinus syndrome; y, years.

**Table 6:** Stroke.

	Population (n)	Age (y), Median or Mean ± SD	Median F-U (y)	Stroke, n (%)	IR Stroke
				Incident Events	
***LMNA *** **mutation carriers**					
Boriani et al. [27], 2003	8	29.5	7	5 (62.5)	8.9
Van Rijsingen et al. [32], 2012	269	36	3.5	N/A	N/A
Anselme et al. [33], 2013	47	38 ± 11	7.9	4 (8.5)	1.1
Van Rijsingen et al. [34], 2013	76	45	42 ± 12 y (mean ± SD)	Arterial TE, 11 (14)	0.3
Kumar et al. [28], 2016	122	41 ± 14	7	10 (8)	1.2
Hasselberg et al. [29], 2018	79	42 ± 16	7.8	N/A	N/A
Nakajima et al. [35], 2018	110 baseline/90 end of f-u	43 ± 15	5	11 (12.2)	2.4
Peretto et al. [36], 2019	164	38	10	N/A	N/A
Ditaranto et al. [37], 2019	40	39	2.5	N/A	N/A
Marchel et al. [38], 2021	15	26	11	N/A	N/A
Barriales-Villa et al. [39], 2021	140	40.4	3.8	Embolism, 14 (10)	2.6
***EMD* mutation carriers**					
Boriani et al. [27], 2003	10	24.5 (affected men)	16	1 (10)	0.6
Marchel et al. [38], 2021	30	21	11	N/A	N/A

Legend: F-U, follow-up; IR, incidence rate; TE, thromboembolic events; y, years.

**Table 7:** Heart failure and heart transplantation.

	Population (n)	Age (y), Median or Mean ± SD	LVEF < 50%, n(%)	LVEF < 45%, n(%)	NYHA ≥ III–IV	Median F-U (y)	NYHA ≥ III–IV	HT, n(%)	IR HT
			Baseline Prevalence				Incident Events or Final Prevalence		
***LMNA *** **mutation carriers**									
Boriani [27], 2003	8	29.5	N/A	N/A	0 (0)	7	1 (12.5)	1 (12.5)	1.3
Van Rijsingen IA [32], 2012	269	36	N/A	89/243 (36.6)	39/260 (15)	3.5	N/A	36 (13.3)	3.8
Anselme [33], 2013	47	38±11	N/A	6 (13)	N/A	7.9	N/A	9 (19)	N/A
Van Rijsingen [34], 2013	76	45	LVEF < 55%, 35(46)	LVEF < 35%, 13(17)	N/A	42 ± 12 (mean ± SD)	N/A	N/A	N/A
Kumar [28], 2016	122	41 ± 14	57 (47)	0 (0)	N/A	7	N/A	10 (8)	0.1
Hasselberg [29], 2018	79	42 ± 16	N/A	29 (36.7)	N/A	7.8	N/A	15 (18)	2.4
Nakajima [35], 2018	110 baseline/90 end of f-U	43 ± 15	22/110 (20)	N/A	8/110 (7.3)	5	30 (34)	N/A	N/A
Peretto [36], 2019	164	38	N/A	5/147 (3.5)	N/A	10	N/A	14 (8.5)	0.9
Ditaranto [37], 2019	40	39	N/A	N/A	7 (17)	2.5	N/A	10 (25)	10
Marchel [38], 2021	15	26	N/A	N/A	0 (0)	11	N/A	N/A	N/A
Barriales-Villa [39], 2021	140	40.4	N/A	53 (37.8)	27 (19.3)	3.8	N/A	29 (20.7)	5.2
***EMD *** **mutation carriers**									
Boriani [27], 2003	10	24.5 (affected males)	N/A	N/A	0 (0)	16	0 (0)	0 (0)	0
Marchel [38], 2021	30	21	N/A	N/A	0 (0)	11	N/A	N/A	N/A

Legend: F-U, follow-up; HT, heart transplantation; IR, incidence rate; LVEF, left ventricular ejection fraction; y, years.

**Table 8:** Mortality.

	Population (n)	Age (y), Median or Mean ± SD	Median F-U (y)	All-Cause Death, n (%)	Cardiac Death, n (%)	IR All-Cause Death	IR Cardiac Death
***LMNA *** **mutation carriers**							
Boriani et al. [27], 2003	8	29.5	7	1 (12.5)	1 (12.5)	1.8	1.8
Van Rijsingen et al. [32], 2012	269	36	3.5	45 (16.7)	41 (15.2)	4.8	4.4
Anselme et al. [33], 2013	47	38 ± 11	7.9	7 (14.8)	4 (8.5)	1.9	1.1
Van Rijsingen et al. [34], 2013	76	45	42 ± 12 (mean ± SD)	N/A	N/A	N/A	N/A
Kumar et al. [28], 2016	122	41 ± 14	7	22 (18)	21 (17.2)	2.6	2.5
Hasselberg et al. [29], 2018	79	42 ± 16	7.8	6 (8)	6 (8)	1	1
Nakajima et al. [35], 2018	110 baseline/90 end of f-U	43 ± 15	5	17 (18.9)	16 (17.7)	3.8	3.6
Peretto et al. [36], 2019	164	38	10	10 (6)	6 (3.6)	0.6	0.4
Ditaranto et al. [37], 2019	40	39	2.5	N/A	N/A	N/A	N/A
Marchel et al. [38], 2021	15	26	11	N/A	N/A	N/A	N/A
Barriales-Villa et al. [39], 2021	140	40.4	3.8	N/A	8 (5.7)	N/A	1.5
***EMD *** **mutation carriers**							
Boriani et al. [27], 2003	10	24.5 (affected males)	16	1 (10)	0 (0)	0.6	0
Marchel et al. [38], 2021	30	21	11	N/A	N/A	N/A	N/A

Legend: IR, incidence rate; y, years.

## Data Availability

Not applicable.

## Supplementary Materials

The following supporting information can be downloaded at https://www.mdpi.com/article/10.3390/biology11040530/s1—Table S1: Full search syntax for systematic review; Table S2: List of studies excluded due to overlapping cohorts.

## References

[1] Emery A.E., Dreifuss F.E. (1966). Unusual type of benign x-linked muscular dystrophy. J. Neurol. Neurosurg. Psychiatry.

[2] Bione S., Maestrini E., Rivella S., Mancini M., Regis S., Romeo G., Toniolo D. (1994). Identification of a novel X-linked gene responsible for Emery-Dreifuss muscular dystrophy. Nat. Genet..

[3] Bonne G., Di Barletta M.R., Varnous S., Bécane H.M., Hammouda E.H., Merlini L., Muntoni F., Greenberg C.R., Gary F., Urtizberea J.A. (1999). Mutations in the gene encoding lamin A/C cause autosomal dominant Emery-Dreifuss muscular dystrophy. Nat. Genet..

[4] Fatkin D., MacRae C., Sasaki T., Wolff M.R., Porcu M., Frenneaux M., Atherton J., Vidaillet H.J., Spudich S., De Girolami U. (1999). Missense mutations in the rod domain of the lamin A/C gene as causes of dilated cardiomyopathy and conduction-system disease. N. Engl. J. Med..

[5] Zhang Q., Bethmann C., Worth N.F., Davies J.D., Wasner C., Feuer A., Ragnauth C.D., Yi Q., Mellad J.A., Warren D.T. (2007). Nesprin-1 and -2 are involved in the pathogenesis of Emery Dreifuss muscular dystrophy and are critical for nuclear envelope integrity. Hum. Mol. Genet..

[6] Zhou C., Li C., Zhou B., Sun H., Koullourou V., Holt I., Puckelwartz M.J., Warren D.T., Hayward R., Lin Z. (2017). Novel nesprin-1 mutations associated with dilated cardiomyopathy cause nuclear envelope disruption and defects in myogenesis. Hum. Mol. Genet..

[7] Meinke P., Mattioli E., Haque F., Antoku S., Columbaro M., Straatman K.R., Worman H.J., Gundersen G.G., Lattanzi G., Wehnert M. (2014). Muscular dystrophy-associated SUN1 and SUN2 variants disrupt nuclear-cytoskeletal connections and myonuclear organization. PLoS Genet..

[8] Gueneau L., Bertrand A.T., Jais J.P., Salih M.A., Stojkovic T., Wehnert M., Hoeltzenbein M., Spuler S., Saitoh S., Verschueren A. (2009). Mutations of the FHL1 gene cause Emery-Dreifuss muscular dystrophy. Am. J. Hum. Genet..

[9] Muchir A., Worman H.J. (2019). Emery-Dreifuss muscular dystrophy: Focal point nuclear envelope. Curr. Opin. Neurol..

[10] Lammerding J., Schulze P.C., Takahashi T., Kozlov S., Sullivan T., Kamm R.D., Stewart C.L., Lee R.T. (2004). Lamin A/C deficiency causes defective nuclear mechanics and mechanotransduction. J. Clin. Investig..

[11] Hutchison C.J. (2002). Lamins: Building blocks or regulators of gene expression?. Nat. Rev. Mol. Cell Biol..

[12] Holt I., Ostlund C., Stewart C.L., Man N., Worman H.J., Morris G.E. (2003). Effect of pathogenic mis-sense mutations in lamin A on its interaction with emerin in vivo. J. Cell Sci..

[13] Cenni V., Squarzoni S., Loi M., Mattioli E., Lattanzi G., Capanni C. (2020). Emerin Phosphorylation during the Early Phase of the Oxidative Stress Response Influences Emerin-BAF Interaction and BAF Nuclear Localization. Cells.

[14] Camozzi D., Capanni C., Cenni V., Mattioli E., Columbaro M., Squarzoni S., Lattanzi G. (2014). Diverse lamin-dependent mechanisms interact to control chromatin dynamics. Focus on laminopathies. Nucleus.

[15] Mattioli E., Columbaro M., Capanni C., Maraldi N.M., Cenni V., Scotlandi K., Marino M.T., Merlini L., Squarzoni S., Lattanzi G. (2011). Prelamin A-mediated recruitment of SUN1 to the nuclear envelope directs nuclear positioning in human muscle. Cell Death Differ..

[16] Roncarati R., Viviani Anselmi C., Krawitz P., Lattanzi G., von Kodolitsch Y., Perrot A., di Pasquale E., Papa L., Portararo P., Columbaro M. (2013). Doubly heterozygous LMNA and TTN mutations revealed by exome sequencing in a severe form of dilated cardiomyopathy. Eur. J. Hum. Genet..

[17] Cenni V., Capanni C., Mattioli E., Schena E., Squarzoni S., Bacalini M.G., Garagnani P., Salvioli S., Franceschi C., Lattanzi G. (2020). Lamin A involvement in ageing processes. Ageing Res. Rev..

[18] Chen S.N., Lombardi R., Karmouch J., Tsai J.Y., Czernuszewicz G., Taylor M.R.G., Mestroni L., Coarfa C., Gurha P., Marian A.J. (2019). DNA Damage Response/TP53 Pathway Is Activated and Contributes to the Pathogenesis of Dilated Cardiomyopathy Associated with LMNA (Lamin A/C) Mutations. Circ. Res..

[19] González J.M., Navarro-Puche A., Casar B., Crespo P., Andrés V. (2008). Fast regulation of AP-1 activity through interaction of lamin A/C, ERK1/2, and c-Fos at the nuclear envelope. J. Cell Biol..

[20] Le Dour C., Macquart C., Sera F., Homma S., Bonne G., Morrow J.P., Worman H.J., Muchir A. (2017). Decreased WNT/β-catenin signalling contributes to the pathogenesis of dilated cardiomyopathy caused by mutations in the lamin a/C gene. Hum. Mol. Genet..

[21] Shin J.Y., Worman H.J. (2022). Molecular Pathology of Laminopathies. Annu. Rev. Pathol..

[22] Sullivan T., Escalante-Alcalde D., Bhatt H., Anver M., Bhat N., Nagashima K., Stewart C.L., Burke B. (1999). Loss of A-type lamin expression compromises nuclear envelope integrity leading to muscular dystrophy. J. Cell Biol..

[23] Fidziańska A., Toniolo D., Hausmanowa-Petrusewicz I. (1998). Ultrastructural abnormality of sarcolemmal nuclei in Emery-Dreifuss muscular dystrophy (EDMD). J. Neurol. Sci..

[24] Nikolova V., Leimena C., McMahon A.C., Tan J.C., Chandar S., Jogia D., Kesteven S.H., Michalicek J., Otway R., Verheyen F. (2004). Defects in nuclear structure and function promote dilated cardiomyopathy in lamin A/C-deficient mice. J. Clin. Investig..

[25] Wong X., Stewart C.L. (2020). The Laminopathies and the Insights They Provide into the Structural and Functional Organization of the Nucleus. Annu. Rev. Genom. Hum. Genet..

[26] Salvarani N., Crasto S., Miragoli M., Bertero A., Paulis M., Kunderfranco P., Serio S., Forni A., Lucarelli C., Dal Ferro M. (2019). The K219T-Lamin mutation induces conduction defects through epigenetic inhibition of SCN5A in human cardiac laminopathy. Nat. Commun..

[27] Boriani G., Gallina M., Merlini L., Bonne G., Toniolo D., Amati S., Biffi M., Martignani C., Frabetti L., Bonvicini M. (2003). Clinical relevance of atrial fibrillation/flutter, stroke, pacemaker implant, and heart failure in Emery-Dreifuss muscular dystrophy: A long-term longitudinal study. Stroke.

[28] Kumar S., Baldinger S.H., Gandjbakhch E., Maury P., Sellal J.M., Androulakis A.F., Waintraub X., Charron P., Rollin A., Richard P. (2016). Long-Term Arrhythmic and Nonarrhythmic Outcomes of Lamin A/C Mutation Carriers. J. Am. Coll. Cardiol..

[29] Hasselberg N.E., Haland T.F., Saberniak J., Brekke P.H., Berge K.E., Leren T.P., Edvardsen T., Haugaa K.H. (2018). Lamin A/C cardiomyopathy: Young onset, high penetrance, and frequent need for heart transplantation. Eur. Heart J..

[30] Page M.J., McKenzie J.E., Bossuyt P.M., Boutron I., Hoffmann T.C., Mulrow C.D., Shamseer L., Tetzlaff J.M., Akl E.A., Brennan S.E. (2021). The PRISMA 2020 statement: An updated guideline for reporting systematic reviews. BMJ.

[31] Stang A. (2010). Critical evaluation of the Newcastle-Ottawa scale for the assessment of the quality of nonrandomized studies in meta-analyses. Eur. J. Epidemiol.

[32] van Rijsingen I.A., Arbustini E., Elliott P.M., Mogensen J., Hermans-van Ast J.F., van der Kooi A.J., van Tintelen J.P., van den Berg M.P., Pilotto A., Pasotti M. (2012). Risk factors for malignant ventricular arrhythmias in lamin a/c mutation carriers a European cohort study. J. Am. Coll. Cardiol..

[33] Anselme F., Moubarak G., Savouré A., Godin B., Borz B., Drouin-Garraud V., Gay A. (2013). Implantable cardioverter-defibrillators in lamin A/C mutation carriers with cardiac conduction disorders. Heart Rhythm.

[34] van Rijsingen I.A., Bakker A., Azim D., Hermans-van Ast J.F., van der Kooi A.J., van Tintelen J.P., van den Berg M.P., Christiaans I., Lekanne Dit Deprez R.H., Wilde A.A. (2013). Lamin A/C mutation is independently associated with an increased risk of arterial and venous thromboembolic complications. Int. J. Cardiol..

[35] Nakajima K., Aiba T., Makiyama T., Nishiuchi S., Ohno S., Kato K., Yamamoto Y., Doi T., Shizuta S., Onoue K. (2018). Clinical Manifestations and Long-Term Mortality in Lamin A/C Mutation Carriers from a Japanese Multicenter Registry. Circ. J..

[36] Peretto G., Di Resta C., Perversi J., Forleo C., Maggi L., Politano L., Barison A., Previtali S.C., Carboni N., Brun F. (2019). Cardiac and Neuromuscular FeatuRes. of Patients with LMNA-Related Cardiomyopathy. Ann. Intern. Med..

[37] Ditaranto R., Boriani G., Biffi M., Lorenzini M., Graziosi M., Ziacchi M., Pasquale F., Vitale G., Berardini A., Rinaldi R. (2019). Differences in cardiac phenotype and natural history of laminopathies with and without neuromuscular onset. Orphanet J. Rare Dis..

[38] Marchel M., Madej-Pilarczyk A., Tymińska A., Steckiewicz R., Ostrowska E., Wysińska J., Russo V., Grabowski M., Opolski G. (2021). Cardiac Arrhythmias in Muscular Dystrophies Associated with Emerinopathy and Laminopathy: A Cohort Study. J. Clin. Med..

[39] Barriales-Villa R., Ochoa J.P., Larrañaga-Moreira J.M., Salazar-Mendiguchía J., Díez-López C., Restrepo-Córdoba M.A., Álvarez-Rubio J., Robles-Mezcua A., Olmo-Conesa M.C., Nicolás-Rocamora E. (2021). Risk predictors in a Spanish cohort with cardiac laminopathies. The REDLAMINA registry. Rev. Esp. Cardiol..

[40] Mitani A.A., Haneuse S. (2020). Small Data Challenges of Studying Rare Diseases. JAMA Netw. Open.

[41] Schnabel R.B., Yin X., Gona P., Larson M.G., Beiser A.S., McManus D.D., Newton-Cheh C., Lubitz S.A., Magnani J.W., Ellinor P.T. (2015). 50 year trends in atrial fibrillation prevalence, incidence, risk factors, and mortality in the Framingham Heart Study: A cohort study. Lancet.

[42] Boriani G., Diemberger I., Martignani C., Biffi M., Branzi A. (2006). The epidemiological burden of atrial fibrillation: A challenge for clinicians and health care systems. Eur. Heart J..

[43] Goette A., Kalman J.M., Aguinaga L., Akar J., Cabrera J.A., Chen S.A., Chugh S.S., Corradi D., D’Avila A., Dobrev D. (2016). EHRA/HRS/APHRS/SOLAECE expert consensus on atrial cardiomyopathies: Definition, characterization, and clinical implication. Europace.

[44] Goette A., Lendeckel U. (2021). Atrial Cardiomyopathy: Pathophysiology and Clinical Consequences. Cells.

[45] van Tintelen J.P., Tio R.A., Kerstjens-Frederikse W.S., van Berlo J.H., Boven L.G., Suurmeijer A.J., White S.J., den Dunnen J.T., te Meerman G.J., Vos Y.J. (2007). Severe myocardial fibrosis caused by a deletion of the 5′ end of the lamin A/C gene. J. Am. Coll. Cardiol..

[46] Eldin A.J., Akinci B., da Rocha A.M., Meral R., Simsir I.Y., Adiyaman S.C., Ozpelit E., Bhave N., Gen R., Yurekli B. (2021). Cardiac phenotype in familial partial lipodystrophy. Clin. Endocrinol..

[47] Boriani G., Vitolo M., Diemberger I., Proietti M., Valenti A.C., Malavasi V.L., Lip G.Y.H. (2021). Optimizing indices of AF susceptibility and burden to evaluate AF severity, risk and outcomes. Cardiovasc. Res..

[48] Gourraud J.B., Khairy P., Abadir S., Tadros R., Cadrin-Tourigny J., Macle L., Dyrda K., Mondesert B., Dubuc M., Guerra P.G. (2018). Atrial fibrillation in young patients. Expert Rev. Cardiovasc. Ther..

[49] Chugh S.S., Havmoeller R., Narayanan K., Singh D., Rienstra M., Benjamin E.J., Gillum R.F., Kim Y.H., McAnulty J.H., Zheng Z.J. (2014). Worldwide epidemiology of atrial fibrillation: A Global Burden of Disease 2010 Study. Circulation.

[50] Steckiewicz R., Stolarz P., Świętoń E., Madej-Pilarczyk A., Grabowski M., Marchel M., Pieniak M., Filipiak K.J., Hausmanowa-Petrusewicz I., Opolski G. (2016). Cardiac pacing in 21 patients with Emery-Dreifuss muscular dystrophy: A single-centre study with a 39-year follow-up. Kardiol. Pol..

[51] Boriani G., Schnabel R.B., Healey J.S., Lopes R.D., Verbiest-van Gurp N., Lobban T., Camm J.A., Freedman B. (2020). Consumer-led screening for atrial fibrillation using consumer-facing wearables, devices and apps: A survey of health care professionals by AF-SCREEN international collaboration. Eur. J. Intern. Med..

[52] Guo Y., Guo J., Shi X., Yao Y., Sun Y., Xia Y., Yu B., Liu T., Chen Y., Lip G.Y.H. (2020). Mobile health technology-supported atrial fibrillation screening and integrated care: A report from the mAFA-II trial Long-term Extension Cohort. Eur. J. Intern. Med..

[53] Yoneda Z.T., Anderson K.C., Quintana J.A., O’Neill M.J., Sims R.A., Glazer A.M., Shaffer C.M., Crawford D.M., Stricker T., Ye F. (2021). Early-Onset Atrial Fibrillation and the Prevalence of Rare Variants in Cardiomyopathy and Arrhythmia Genes. JAMA Cardiol..

[54] Schnabel R.B., Haeusler K.G., Healey J.S., Freedman B., Boriani G., Brachmann J., Brandes A., Bustamante A., Casadei B., Crijns H.J.G.M. (2019). Searching for Atrial Fibrillation Poststroke: A White Paper of the AF-SCREEN International Collaboration. Circulation.

[55] Hindricks G., Potpara T., Dagres N., Arbelo E., Bax J.J., Blomström-Lundqvist C., Boriani G., Castella M., Dan G.A., Dilaveris P.E. (2020). 2020 ESC Guidelines for the diagnosis and management of atrial fibrillation developed in collaboration with the European Association of Cardio-Thoracic Surgery (EACTS). Eur. Heart J..

[56] Boriani G., Vitolo M., Lane D.A., Potpara T.S., Lip G.Y. (2021). Beyond the 2020 guidelines on atrial fibrillation of the European society of cardiology. Eur. J. Intern Med..

[57] Boriani G., Biagini E., Ziacchi M., Malavasi V.L., Vitolo M., Talarico M., Mauro E., Gorlato G., Lattanzi G. (2018). Cardiolaminopathies from bench to bedside: Challenges in clinical decision-making with focus on arrhythmia-related outcomes. Nucleus.

[58] Meune C., Van Berlo J.H., Anselme F., Bonne G., Pinto Y.M., Duboc D. (2006). Primary prevention of sudden death in patients with lamin A/C gene mutations. N. Engl. J. Med..

[59] van Berlo J.H., de Voogt W.G., van der Kooi A.J., van Tintelen J.P., Bonne G., Yaou R.B., Duboc D., Rossenbacker T., Heidbüchel H., de Visser M. (2005). Meta-analysis of clinical characteristics of 299 carriers of LMNA gene mutations: Do lamin A/C mutations portend a hig5757h risk of sudden death?. J. Mol. Med..

[60] Glikson M., Nielsen J.C., Kronborg M.B., Michowitz Y., Auricchio A., Barbash I.M., Barrabés J.A., Boriani G., Braunschweig F., Brignole M. (2021). 2021 ESC Guidelines on cardiac pacing and cardiac resynchronization therapy. Eur. Heart J..

[61] Køber L., Thune J.J., Nielsen J.C., Haarbo J., Videbæk L., Korup E., Jensen G., Hildebrandt P., Steffensen F.H., Bruun N.E. (2016). Defibrillator Implantation in Patients with Nonischemic Systolic Heart Failure. N. Engl. J. Med..

[62] Gatzoulis K.A., Vouliotis A.I., Tsiachris D., Salourou M., Archontakis S., Dilaveris P., Gialernios T., Arsenos P., Karystinos G., Sideris S. (2013). Primary prevention of sudden cardiac death in a nonischemic dilated cardiomyopathy population: Reappraisal of the role of programmed ventricular stimulation. Circ. Arrhythm. Electrophysiol..

[63] Escobar-Lopez L., Ochoa J.P., Mirelis J.G., Espinosa M., Navarro M., Gallego-Delgado M., Barriales-Villa R., Robles-Mezcua A., Basurte-Elorz M.T., Gutiérrez García-Moreno L. (2021). Association of Genet. ic Variants with Outcomes in Patients with Nonischemic Dilated Cardiomyopathy. J. Am. Coll. Cardiol..

[64] Wahbi K., Ben Yaou R., Gandjbakhch E., Anselme F., Gossios T., Lakdawala N.K., Stalens C., Sacher F., Babuty D., Trochu J.N. (2019). Development and Validation of a New Risk Prediction Score for Life-Threatening Ventricular Tachyarrhythmias in Laminopathies. Circulation.

[65] Kayvanpour E., Sedaghat-Hamedani F., Amr A., Lai A., Haas J., Holzer D.B., Frese K.S., Keller A., Jensen K., Katus H.A. (2017). Genotype-phenotype associations in dilated cardiomyopathy: Meta-analysis on more than 8000 individuals. Clin. Res. Cardiol..

[66] Taylor M.R., Fain P.R., Sinagra G., Robinson M.L., Robertson A.D., Carniel E., Di Lenarda A., Bohlmeyer T.J., Ferguson D.A., Brodsky G.L. (2003). Natural history of dilated cardiomyopathy due to lamin A/C gene mutations. J. Am. Coll. Cardiol..

[67] Sanna T., Dello Russo A., Toniolo D., Vytopil M., Pelargonio G., De Martino G., Ricci E., Silvestri G., Giglio V., Messano L. (2003). Cardiac featuRes. of Emery-Dreifuss muscular dystrophy caused by lamin A/C gene mutations. Eur. Heart J..

[68] Gigli M., Merlo M., Graw S.L., Barbati G., Rowland T.J., Slavov D.B., Stolfo D., Haywood M.E., Dal Ferro M., Altinier A. (2019). Genet. ic Risk of Arrhythmic Phenotypes in Patients with Dilated Cardiomyopathy. J. Am. Coll. Cardiol..

[69] Atalaia A., Ben Yaou R., Wahbi K., De Sandre-Giovannoli A., Vigouroux C., Bonne G. (2021). Laminopathies’ Treatments Systematic Review: A Contribution Towards a ‘Treatabolome’. J. Neuromuscul. Dis..

