# Supplementary material for: Clinical Profile, Arrhythmias, and Adverse Cardiac Outcomes in Emery–Dreifuss Muscular Dystrophies: A Systematic Review of the Literature
Source: Biology (Basel). 2022 Mar 30;11(4):530. doi: 10.3390/biology11040530 (PMC9031530; doi:10.3390/biology11040530)
Supplement: Supplementary file 1 [file biology-11-00530-s001.zip › biology-1645697-supplementary.pdf]

Supplementary material

Table S1.

Full search syntax for systematic review:
(("Muscular Dystrophy, Emery-Dreifuss"[Mesh]) OR ("Emery-Dreifuss") OR ("LMNA") OR ("Lamin A/C") OR ("Laminopathies"[Mesh]) OR ("Muscular Dystrophies") OR ("lamin") OR ("Laminopathy")) AND (("Atrial Fibrillation"[Mesh]) OR ("atrial fibrillation") OR ("Defibrillators, Implantable"[Mesh]) OR ("defibrillator") OR ("Pacemaker, Artificial"[Mesh]) OR ("Pacemaker") OR ("Arrhythmias, Cardiac"[Mesh]) OR ("Heart Failure"[Mesh]) OR ("Arrhythmias") OR ("Heart Failure") OR ("Death, Sudden, Cardiac"[Mesh]))

Table S2.

List of studies excluded due to overlapping cohorts
Hasselberg NE, Edvardsen T, Petri H, Berge KE, Leren TP, Bundgaard H, Haugaa KH. Risk prediction of ventricular arrhythmias and myocardial function in Lamin A/C mutation positive subjects. Europace. 2014 Apr;16(4):563-71. doi: 10.1093/europace/eut291. Epub 2013 Sep 20. PMID: 24058181. (72)
van Rijsingen IA, Nannenberg EA, Arbustini E, Elliott PM, Mogensen J, Hermans-van Ast JF, van der Kooi AJ, van Tintelen JP, van den Berg MP, Grasso M, Serio A, Jenkins S, Rowland C, Richard P, Wilde AA, Perrot A, Pankuweit S, Zwinderman AH, Charron P, Christiaans I, Pinto YM. Gender-specific differences in major cardiac events and mortality in lamin A/C mutation carriers. Eur J Heart Fail. 2013 Apr;15(4):376-84. doi: 10.1093/eurjhf/hfs191. Epub 2012 Nov 25. PMID: 23183350.(73)
Pasotti M, Klersy C, Pilotto A, Marziliano N, Rapezzi C, Serio A, Mannarino S, Gambarin F, Favalli V, Grasso M, Agozzino M, Campana C, Gavazzi A, Febo O, Marini M, Landolina M, Mortara A, Piccolo G, Viganò M, Tavazzi L, Arbustini E. Long-term outcome and risk stratification in dilated cardiolaminopathies. J Am Coll Cardiol. 2008 Oct 7;52(15):1250-60. doi: 10.1016/j.jacc.2008.06.044. PMID: 18926329. (74)

Nishiuchi S, Makiyama T, Aiba T, Nakajima K, Hirose S, Kohjitani H, Yamamoto Y, Harita T, Hayano M, Wuriyanghai Y, Chen J, Sasaki K, Yagihara N, Ishikawa T, Onoue K, Murakoshi N, Watanabe I, Ohkubo K, Watanabe H, Ohno S, Doi T, Shizuta S, Minamino T, Saito Y, Oginosawa Y, Nogami A, Aonuma K, Kusano K, Makita N, Shimizu W, Horie M, Kimura T. Gene-Based Risk Stratification for Cardiac Disorders in *LMNA* Mutation Carriers. Circ Cardiovasc Genet. 2017 Dec;10(6):e001603. doi: 10.1161/CIRCGENETICS.116.001603. PMID: 29237675.(75)
Maggi L, D'Amico A, Pini A, Sivo S, Pane M, Ricci G, Vercelli L, D'Ambrosio P, Travaglini L, Sala S, Brenna G, Kapetis D, Scarlato M, Pegoraro E, Ferrari M, Toscano A, Benedetti S, Bernasconi P, Colleoni L, Lattanzi G, Bertini E, Mercuri E, Siciliano G, Rodolico C, Mongini T, Politano L, Previtali SC, Carboni N, Mantegazza R, Morandi L. LMNA-associated myopathies: the Italian experience in a large cohort of patients. Neurology. 2014 Oct 28;83(18):1634-44. doi: 10.1212/WNL.0000000000000934. Epub 2014 Oct 1. PMID: 25274841.(76)
